# Hispanics or Latinos Living with Diagnosed HIV: Progress Along the Continuum of HIV Care — United States, 2010

**Published:** 2014-10-10

**Authors:** Zanetta Gant, Heather Bradley, Xiaohong Hu, Jacek Skarbinski, H. Irene Hall, Amy Lansky

**Affiliations:** 1 Division of HIV/AIDS Prevention, National Center for HIV/AIDS, Viral Hepatitis, STD, and TB Prevention, CDC

The goals of the National HIV/AIDS Strategy are to reduce new human immunodeficiency virus (HIV) infections, increase access to care and improve health outcomes for persons living with HIV, and reduce HIV-related health disparities (1). In July 2013, by presidential executive order, the HIV Care Continuum Initiative was established, focusing on accelerating federal efforts to increase HIV testing, care, and treatment (2). Hispanics or Latinos* are disproportionately affected by HIV infection; the annual rate of HIV diagnosis among Hispanics or Latinos is approximately three times that of non-Hispanic whites (3). To achieve the goals of the National HIV/AIDS Strategy, and to be consistent with the HIV Care Continuum Initiative, Hispanics or Latinos living with HIV infection need improved levels of care and viral suppression (4–6). Achieving these goals calls for 85% of Hispanics or Latinos with diagnosed HIV to be linked to care, 80% to be retained in care, and the proportion with an undetectable viral load (VL) to increase 20% by 2015 (1). Analysis of data from the National HIV Surveillance System (NHSS)† and the Medical Monitoring Project (MMP)§ regarding progress along the HIV care continuum during 2010 for Hispanics or Latinos with diagnosed HIV infection indicated that 80.3% of HIV-diagnosed Hispanics or Latinos were linked to care, 54.4% were retained in care, 44.4% were prescribed antiretroviral therapy (ART), and 36.9% had achieved viral suppression (VL result of ≤200 copies/mL). Among Hispanic or Latino males and females, the percentages that were linked to care, were prescribed ART, and had achieved viral suppression were similar; however, the percentage retained in care was lower among males compared with females. The levels of linkage to care and viral suppression were lower among Hispanics or Latinos with HIV infection attributed to injection drug use than among those with HIV infection attributed to heterosexual or male-to-male sexual contact. These data demonstrate the need for implementation of interventions and public health strategies that increase linkage to care, retention in care, and consistent ART among Hispanics or Latinos, particularly Hispanics or Latinos who inject drugs.

Data from NHSS for 2010 reported to CDC through December 2012 were used to determine the numbers of Hispanics or Latinos aged ≥13 years newly diagnosed with HIV and living with diagnosed HIV and the numbers and percentages linked to care and retained in care. Nineteen jurisdictions met the criteria for the collection and reporting of CD4+ T-lymphocyte (CD4) and VL test results,¶ which are the data needed to assess linkage and retention in care. Linkage to care** was calculated among Hispanics or Latinos with new HIV diagnoses during 2010 who resided in any of the 19 jurisdictions at diagnosis. Retention in care†† was assessed among Hispanics or Latinos with HIV diagnosed by December 31, 2009, who resided in any of the 19 jurisdictions at the time of diagnosis, and were alive on December 31, 2010, (i.e., persons living with diagnosed HIV). Data were statistically adjusted for missing HIV transmission categories (3).

Data from MMP were used to estimate ART prescription§§ and viral suppression¶¶ among Hispanics or Latinos aged ≥18 years using methods that have been described previously (5). The MMP values are weighted national estimates of the numbers of Hispanics or Latinos who received medical care during January–April 2010 and had documentation of ART prescription and viral suppression. Percentages were calculated among Hispanics or Latinos whose HIV infection was diagnosed by December 31, 2009, and who were alive on December 31, 2010, in the United States and Puerto Rico (denominators were based on NHSS data). Data analyses were limited to 2010, the most recent year data were available for persons living with HIV infection.

Of the 2,992 Hispanics or Latinos with HIV infection diagnosed during 2010 in the 19 jurisdictions, 2,402 (80.3%) were linked to care ≤3 months after HIV diagnosis (Table 1). Among males and females, 80.2% and 80.7%, respectively, were linked to care. The percentage of linkage to care was similar across age categories, with persons aged 13–24 years having the lowest percentage linked to care (78.7%) and persons aged 45–54 years having the highest percentage linked to care (81.9%). By transmission category, the lowest percentage of linkage to care was among males and females with infection attributed to injection drug use (76.5% and 78.6%, respectively), whereas the highest percentage of linkage to care was among males and females with infection attributed to heterosexual contact (82.9% and 81.0%, respectively).

Among 70,213 Hispanics or Latinos aged ≥13 years residing in the 19 jurisdictions at HIV diagnosis and reported living at the end of 2010, 54.4% were retained in care (Table 2). Of these, males (52.7%) had a 7% lower percentage retained in care compared with females (59.7%). By age group, the percentage retained in care was similar, with persons aged 25–34 years having the lowest percentage retained in care (52.2%) and persons aged 45–54 years having the highest percentage retained in care (55.7%). By transmission category, the lowest percentage retained in care was among males with infection attributed to injection drug use (47.6%), and the highest percentage was among females with infection attributed to heterosexual contact (59.8%).

Of 172,536 Hispanics or Latinos aged ≥18 years living with diagnosed HIV on December 31, 2010, in the United States and Puerto Rico, 76,650 (44.4%) were prescribed ART (Table 3). Among males and females, 44.0% and 45.7%, respectively, were prescribed ART. Prevalence of ART prescription was lowest among those aged 25–34 years (36.7%) and highest among those aged ≥55 years (59.3%). The lowest percentage of ART prescription by transmission category was among males with infection attributed to injection drug use (31.0%), and the highest percentage was among females with infection attributed to heterosexual contact (49.8%).

Of Hispanics or Latinos living with diagnosed HIV in the United States and Puerto Rico, 36.9% had achieved viral suppression at their most recent test. Males and females had nearly the same percentage of viral suppression (36.9% and 37.0%, respectively). Persons aged 25–34 years had the lowest percentage of viral suppression (28.6%), and persons aged ≥55 years had the highest percentage (54.3%). By transmission category, females with infection attributed to injection drug use had the lowest percentage of viral suppression (23.4%), whereas females with infection attributed to heterosexual contact had the highest percentage (42.6%).

## Discussion

The results of the analysis described in this report indicate that, in 2010, among adult and adolescent Hispanics or Latinos of all age groups and both sexes who were diagnosed with HIV, 80.3% were linked to care, 54.4% were retained in care, 44.4% were prescribed ART, and 36.9% had achieved viral suppression. Across the HIV care continuum, Hispanics or Latinos have higher percentages of linkage to and retention in care and ART prescription compared with the national population of persons with HIV, but they have a lower percentage of viral suppression compared with the same national population (4). Among Hispanics or Latinos, percentages of linkage to and retention in care are similar across age groups; this similarity by age is not observed among the national population of persons with HIV or among blacks or African Americans with HIV (4).

Hispanics or Latinos with HIV infection might not seek, receive, or adhere to HIV care or achieve viral suppression for reasons including lack of health insurance, language barriers, geographic differences, and migration patterns (7,8). HIV programs that focus on care and treatment for Hispanics or Latinos might strengthen efforts to link and retain persons with HIV in care and promote adherence to medication to achieve optimal health outcomes. Evidence-based interventions with demonstrated efficacy in scientific studies and effectiveness in practice settings also might be considered (9).

Hispanics or Latinos with HIV infection attributed to injection drug use or male-to-male sexual contact and injection drug use typically had lower levels of linkage to care, retention in care, ART prescription, and viral suppression than those with HIV infection attributed to heterosexual or male-to-male sexual contact. In addition to interventions to ensure that all persons with HIV infection receive optimal care to improve health outcomes, targeted strategies for Hispanics or Latinos who inject drugs might be needed to achieve improvements at each step of the continuum. Providing comprehensive prevention services and referrals to persons who inject drugs, such as those offered by many syringe exchange programs, can help reduce the spread of HIV. These programs can also serve as gateways to care and treatment for HIV infection, thus serving as an effective public health approach for this population (10).

The findings in this report are subject to at least two limitations. First, analyses based on NHSS data are limited to 19 jurisdictions with complete reporting of all levels of CD4 and VL test results; data from these areas represent approximately 45% of all Hispanics or Latinos living with diagnosed HIV on December 31, 2010, in the United States, and might not be representative of all Hispanics or Latinos in the United States. Second, certain analyses in this study are based on different populations, and the results cannot be compared because linkage to care and retention in care were based on data for persons aged ≥13 years from 19 jurisdictions, whereas ART prescription and viral suppression were based on weighted estimates of persons receiving care who were aged ≥18 years from the United States and Puerto Rico.

CDC and its partners are pursuing a high-impact prevention*** approach to advance the goals of the National HIV/AIDS Strategy and maximize the effectiveness of current HIV prevention and care methods. Testing is a critical first step of entry into the HIV continuum of care. CDC supports HIV testing projects and campaigns that focus on Hispanics or Latinos. One such campaign is Reasons (Razones),††† which is the agency’s first national effort to encourage HIV testing among Latino gay and bisexual men, who comprise the majority of Hispanics or Latinos diagnosed with HIV. CDC also supports multiple projects to optimize outcomes along the continuum of care, such as the HIV Screening. Standard Care. Testing and Linking African American and Hispanic/Latino Patients to Care.§§§ campaign, which is a new segment of the Act Against AIDS campaign tailored to help health care providers improve HIV outcomes among African American and Hispanic or Latino patients by making HIV testing and linking to care the clinical standard. Another project is the Care and Prevention in the United States¶¶¶ demonstration project, which seeks to increase linkage to, retention in, and reengagement in care for all persons with HIV, including racial and ethnic minorities, with the goal of reducing HIV-related morbidity and mortality by addressing social, economic, clinical, and structural factors influencing HIV health outcomes. The results of the analyses described in this report underscore the need for enhanced linkage to care, retention in care, and viral suppression for Hispanics or Latinos. Focusing prevention and care efforts on populations that bear a disproportionate burden of HIV disease could lead to reductions in HIV incidence and health inequities and help achieve the goals of the National HIV/AIDS Strategy.

What is already known on this topic?

The 2010 annual rate of human immunodeficiency virus (HIV) diagnosis among Hispanics or Latinos was approximately three times that of non-Hispanic whites. The percentages of Hispanics or Latinos linked to care, retained in care, taking antiretroviral medications, and achieving viral suppression have been lower than those for whites but higher than for blacks or African Americans.

What is added by this report?

Data from 2010 indicate that 80.3% of HIV-infected Hispanics or Latinos were linked to care, 54.4% were retained in care, 44.4% were prescribed antiretroviral therapy (ART), and 36.9% had achieved viral suppression. Among Hispanic or Latino males and females, the percentages that were linked to care, were prescribed ART, and had achieved viral suppression were similar; however, the percentage retained in care was lower among males compared with females. The levels of linkage to care and viral suppression were lower among Hispanics or Latinos with HIV infection attributed to injection drug use than among those with HIV infection attributed to heterosexual or male-to-male sexual contact.

What are the implications for public health practice?

Increasing the proportion of Hispanics or Latinos living with HIV who are receiving care is critical for achieving the goals of the National HIV/AIDS Strategy to reduce new infections, improve health outcomes, and decrease health disparities. Among Hispanics or Latinos, targeted strategies for different groups, such as persons who inject drugs, might be needed to achieve improvements at each step of the HIV care continuum.

## Figures and Tables

**TABLE 1:** Linkage to HIV medical care within 3 months after HIV diagnosis* among Hispanics/Latinos aged ≥13 years, by selected characteristics — National HIV Surveillance System, 19 jurisdictions,† United States, 2010

Characteristic	No. of HIV diagnoses	Linkage to care§	

		No.	(%)
**Sex**			
Male	2,499	2,004	(80.2)
Female	493	398	(80.7)
**Age group at diagnosis (yrs)**			
13–24	583	459	(78.7)
25–34	1,031	817	(79.2)
35–44	775	633	(81.7)
45–54	420	344	(81.9)
≥55	183	149	(81.4)
**Transmission category** ¶			
Male-to-male sexual contact	2,060	1,653	(80.3)
Injection drug use			
Male	172	132	(76.5)
Female	64	50	(78.6)
Male-to-male sexual contact and injection drug use	86	69	(79.4)
Heterosexual contact**			
Male	178	148	(82.9)
Female	428	347	(81.0)
**Total** ††	**2,992**	**2,402**	(**80.3**)

**Abbreviation:** HIV = human immunodeficiency virus.

* Data include persons with a diagnosis of HIV infection regardless of stage of disease at diagnosis. Hispanics/Latinos can be of any race.

† The 19 jurisdictions were California (Los Angeles County and San Francisco only), Delaware, District of Columbia, Georgia, Hawaii, Illinois, Indiana, Iowa, Louisiana, Michigan, Minnesota, Missouri, Nebraska, New Hampshire, New York, North Dakota, South Carolina, West Virginia, and Wyoming.

§ One or more CD4+ T-lymphocyte or viral load test within 3 months after HIV diagnosis.

¶ Data statistically adjusted to account for missing transmission categories.

** Heterosexual contact with a person known to have, or to be at high risk for, HIV infection.

†† Includes three persons with diagnosed infection attributed to hemophilia, blood transfusion, perinatal exposure, and risk factor not reported or not identified.

**TABLE 2:** Retention in HIV medical care among Hispanics/Latinos aged ≥13 years with HIV infection diagnosed by December 31, 2009,* who were alive on December 31, 2010, by selected characteristics — National HIV Surveillance System, 19 jurisdictions,† United States

Characteristic	No.	Retention in care in 2010§	

		No.	(%)
**Sex**			
Male	53,918	28,434	(52.7)
Female	16,295	9,735	(59.7)
**Age group on December 31, 2009 (yrs)**			
13–24	2,880	1,592	(55.3)
25–34	10,447	5,449	(52.2)
35–44	21,778	11,823	(54.3)
45–54	23,277	12,955	(55.7)
≥55	11,831	6,350	(53.7)
**Transmission category** ¶			
Male-to-male sexual contact	34,254	18,515	(54.1)
Injection drug use			
Male	11,060	5,263	(47.6)
Female	4,980	2,952	(59.3)
Male-to-male sexual contact and injection drug use	3,669	2,043	(55.7)
Heterosexual contact**			
Male	4,266	2,256	(52.9)
Female	10,670	6,378	(59.8)
Other††			
Male	668	356	(53.3)
Female	645	404	(62.7)
**Total**	**70,213**	**38,169**	(**54.4**)

**Abbreviation:** HIV = human immunodeficiency virus.

* Data include persons with a diagnosis of HIV infection regardless of stage of disease at diagnosis. Hispanics/Latinos can be of any race.

† The 19 jurisdictions were California (Los Angeles County and San Francisco only), Delaware, District of Columbia, Georgia, Hawaii, Illinois, Indiana, Iowa, Louisiana, Michigan, Minnesota, Missouri, Nebraska, New Hampshire, New York, North Dakota, South Carolina, West Virginia, and Wyoming.

§ Two or more CD4+ T-lymphocyte or viral load tests performed at least 3 months apart during 2010.

¶ Data statistically adjusted to account for missing transmission categories.

** Heterosexual contact with a person known to have, or to be at high risk for, HIV infection.

†† Includes persons with diagnosed infection attributed to hemophilia, blood transfusion, perinatal exposure, and risk factor not reported or not identified.

**TABLE 3:** Prescription of antiretroviral therapy (ART) and viral suppression among Hispanics/Latinos aged ≥18 years with HIV infection diagnosed by December 31, 2009,* who were alive on December 31, 2010, by selected characteristics — National HIV Surveillance System, Medical Monitoring Project, United States and Puerto Rico

Characteristic	No.†	ART prescription§		Viral suppression¶	
	
		No.	(%)	No.	(%)
**Sex**					
Male	133,209	58,590	(44.0)	49,184	(36.9)
Female	39,327	17,963	(45.7)	14,561	(37.0)
**Age group at interview (yrs)**					
18–24	6,182	2,684	(43.4)	1,884	(30.5)
25–34	28,747	10,555	(36.7)	8,224	(28.6)
35–44	55,998	23,553	(42.1)	19,201	(34.3)
45–54	55,644	24,471	(44.0)	20,350	(36.6)
≥55	25,965	15,387	(59.3)	14,087	(54.3)
**Transmission category** **					
Male-to-male sexual contact	82,410	40,509	(49.2)	34,233	(41.5)
Injection drug use					
Male	26,545	8,241	(31.0)	7,379	(27.8)
Female	10,312	3,516	(34.1)	2,410	(23.4)
Male-to-male sexual contact and injection drug use	9,082	3,041	(33.5)	2,514	(27.7)
Heterosexual contact††					
Male	14,159	6,505	(45.9)	5,009	(35.4)
Female	28,173	14,019	(49.8)	12,014	(42.6)
Other transmission§§	1,855	819	(44.2)	186	(10.0)
**Total** ¶¶	**172,536**	**76,650**	(**44.4**)	**63,745**	(**36.9**)

**Abbreviation:** HIV = human immunodeficiency virus.

* Data include persons with a diagnosis of HIV infection regardless of stage of disease at diagnosis. Hispanics/Latinos can be of any race.

† National HIV Surveillance System estimates for United States and Puerto Rico.

§ Medical Monitoring Project estimates for United States and Puerto Rico for persons who received medical care during January–April 2010 and who had documentation of ART prescription in the medical record.

¶ Medical Monitoring Project estimates for United States and Puerto Rico for persons who received medical care during January–April 2010 and whose most recent HIV viral load in the preceding 12 months was undetectable or ≤200 copies/mL.

** Data statistically adjusted to account for missing transmission categories.

†† Heterosexual contact with a person known to have, or to be at high risk for, HIV infection.

§§ Includes persons with diagnosed infection attributed to hemophilia, blood transfusion, perinatal exposure, or risk factor not reported or not identified.

¶¶ Estimates might not sum to total.

## Footnotes

* Hispanics or Latinos can be of any race.

† NHSS is the primary source for monitoring HIV trends in the United States. The system collects, analyzes, and disseminates information about new and existing cases of HIV infection.

§ MMP is a supplemental HIV surveillance system designed to produce nationally representative estimates of the prevalence of behavioral and clinical characteristics among HIV-infected adults aged ≥18 years receiving medical care in the United States and Puerto Rico.

¶ The 19 jurisdictions were California (Los Angeles County and San Francisco only), Delaware, District of Columbia, Georgia, Hawaii, Illinois, Indiana, Iowa, Louisiana, Michigan, Minnesota, Missouri, Nebraska, New Hampshire, New York, North Dakota, South Carolina, West Virginia, and Wyoming. The criteria for complete reporting were as follows: 1) the jurisdiction’s laws or regulations required reporting of all CD4 and VL test results to the state or local health department, 2) ≥95% of all laboratory test results were reported by laboratories that conduct HIV-related testing for each jurisdiction, and 3) the jurisdiction reported to CDC all CD4 and VL results received since at least January 2010.

** Defined as having one or more CD4 (count or percentage) or VL test performed within 3 months after HIV diagnosis during 2010, including those performed during the same month as diagnosis.

†† Defined as having two or more CD4 or VL results at least 3 months apart during 2010, among persons diagnosed through December 31, 2009, and alive on December 31, 2010.

§§ ART prescription was based on MMP data for all Hispanic or Latino MMP participants in the 2010 data collection cycle.

¶¶ Viral suppression was based on all Hispanic or Latino MMP participants in the 2010 data collection cycle and was defined as having a VL result of ≤200 copies/mL at the most recent HIV VL in the preceding 12 months. The cut-off value of ≤200 copies/mL was based on the U.S. Department of Health and Human Services recommended definition of virologic failure.

*** Additional information available at http://www.cdc.gov/nchhstp/newsroom/hivfactsheets/future/high-impact-prevention.htm.

††† Additional information available at http://hivtest.cdc.gov/reasons.

§§§ Additional information available at http://www.cdc.gov/actagainstaids/campaigns/hssc/index.html.

¶¶¶ Additional information available at http://www.cdc.gov/hiv/prevention/demonstration/capus.
